# Loss of conserved long non-coding RNA MIR503HG leads to altered NOTCH pathway signalling and left ventricular non-compaction cardiomyopathy

**DOI:** 10.1093/cvr/cvaf043

**Published:** 2025-04-04

**Authors:** João P Monteiro, Diptarka Saha, Ana-Mishel Spiroski, Saiba Mahesh, Peter Kaltzis, Abhijit Nadavallil, Vaibhao Janbandhu, Nicholas J Murray, Francesco Severi, Azzurra Laura De Pace, Sandra Sánchez-Esteban, Julie Rodor, Abdelaziz Beqqali, Matthew Bennett, Kevin Stewart, Adrian Thomson, Patrick W F Hadoke, Dyana Markose, John R Wilson-Kanamori, Neil C Henderson, Dónal O’Carroll, Thomas Quertermous, Richard P Harvey, Gonzalo del Monte-Nieto, Andrew H Baker

**Affiliations:** Centre for Cardiovascular Science, Queen’s Medical Research Institute, University of Edinburgh, 47 Little France Crescent, Edinburgh EH16 4TJ, UK; Division of Cardiovascular Medicine, Stanford University School of Medicine, Stanford, CA 94305, USA; Australian Regenerative Medicine Institute, 15 Innovation Walk, Monash University, Clayton, VIC 3800, Australia; Centre for Cardiovascular Science, Queen’s Medical Research Institute, University of Edinburgh, 47 Little France Crescent, Edinburgh EH16 4TJ, UK; Australian Regenerative Medicine Institute, 15 Innovation Walk, Monash University, Clayton, VIC 3800, Australia; Australian Regenerative Medicine Institute, 15 Innovation Walk, Monash University, Clayton, VIC 3800, Australia; Australian Regenerative Medicine Institute, 15 Innovation Walk, Monash University, Clayton, VIC 3800, Australia; Victor Chang Cardiac Research Institute, Darlinghurst, NSW 2010, Australia; School of Clinical Medicine, UNSW Sydney, Kensington, NSW 2052, Australia; Victor Chang Cardiac Research Institute, Darlinghurst, NSW 2010, Australia; School of Clinical Medicine, UNSW Sydney, Kensington, NSW 2052, Australia; Centre for Regenerative Medicine, Institute for Regeneration and Repair, University of Edinburgh, Edinburgh EH16 4UU, UK; Centre for Regenerative Medicine, Institute for Regeneration and Repair, University of Edinburgh, Edinburgh EH16 4UU, UK; Centre for Cardiovascular Science, Queen’s Medical Research Institute, University of Edinburgh, 47 Little France Crescent, Edinburgh EH16 4TJ, UK; Centre for Cardiovascular Science, Queen’s Medical Research Institute, University of Edinburgh, 47 Little France Crescent, Edinburgh EH16 4TJ, UK; Centre for Cardiovascular Science, Queen’s Medical Research Institute, University of Edinburgh, 47 Little France Crescent, Edinburgh EH16 4TJ, UK; Centre for Cardiovascular Science, Queen’s Medical Research Institute, University of Edinburgh, 47 Little France Crescent, Edinburgh EH16 4TJ, UK; Centre for Cardiovascular Science, Queen’s Medical Research Institute, University of Edinburgh, 47 Little France Crescent, Edinburgh EH16 4TJ, UK; Centre for Cardiovascular Science, Queen’s Medical Research Institute, University of Edinburgh, 47 Little France Crescent, Edinburgh EH16 4TJ, UK; Centre for Cardiovascular Science, Queen’s Medical Research Institute, University of Edinburgh, 47 Little France Crescent, Edinburgh EH16 4TJ, UK; Centre for Inflammation Research, Institute for Regeneration and Repair, University of Edinburgh, Edinburgh EH16 4UU, UK; Centre for Inflammation Research, Institute for Regeneration and Repair, University of Edinburgh, Edinburgh EH16 4UU, UK; Centre for Inflammation Research, Institute for Regeneration and Repair, University of Edinburgh, Edinburgh EH16 4UU, UK; MRC Human Genetics Unit, Institute of Genetics and Cancer, University of Edinburgh, Edinburgh EH4 2XU, UK; Centre for Regenerative Medicine, Institute for Regeneration and Repair, University of Edinburgh, Edinburgh EH16 4UU, UK; Division of Cardiovascular Medicine, Stanford University School of Medicine, Stanford, CA 94305, USA; Victor Chang Cardiac Research Institute, Darlinghurst, NSW 2010, Australia; School of Clinical Medicine, UNSW Sydney, Kensington, NSW 2052, Australia; School of Biotechnology and Biomolecular Science, UNSW Sydney, Kensington, NSW 2052, Australia; Australian Regenerative Medicine Institute, 15 Innovation Walk, Monash University, Clayton, VIC 3800, Australia; Centre for Cardiovascular Science, Queen’s Medical Research Institute, University of Edinburgh, 47 Little France Crescent, Edinburgh EH16 4TJ, UK; Department of Pathology, Cardiovascular Research Institute Maastricht, Maastricht University Medical Center, 6229 HX Maastricht, The Netherlands

**Keywords:** Long non-coding RNA, Left ventricular non-compaction, Heart development

## Abstract

**Aims:**

The highly conserved long non-coding RNA (lncRNA) MIR505HG has been primarily recognized as a precursor for microRNAs (miR)-424 and miR-503. However, studies have since demonstrated that MIR503HG has distinct functions from its associated miRNAs, playing important roles in cell proliferation, invasion, apoptosis, and differentiation. While these miRNAs are known to influence cardiomyocyte differentiation, the specific role of MIR503HG in heart development remains unexplored. We seek to determine how MIR503HG deletion impacts ventricular chamber development and to identify underlying molecular mechanisms.

**Methods and results:**

To study the role of the lncRNA *in vivo*, we generated a functional MIR503HG knockout mouse model (*MIR503HG^−/−^*) using a synthetic polyadenylation signal to terminate MIR503HG transcription without affecting miR-424/503 expression. We performed morphological analyses on embryonic and adult hearts using microCT along with cardiac functional analysis via transthoracic echocardiography. We further apply single-nuclei RNA sequencing (snRNA-seq) on adult hearts to identify potential molecular mechanisms underlying the observed phenotypes. Functional deletion of MIR503HG alone was associated with reduced compact myocardium thickness and increased trabecular myocardium in the left ventricle (LV) at embryonic day 17.5 compared to wild-type mice, indicating a LV non-compaction (LVNC) phenotype. Moreover, adult *MIR503HG^−/−^* mutant hearts showed increased trabecular complexity, impaired LV relaxation, and mitral valve regurgitation. SnRNA-seq further revealed altered expression of several genes associated with cardiomyocyte function and LVNC, including *Actc1*, *Mib1*, *Mybpc3*, and *Myh7*. Lastly, Notch1 activity was also significantly increased in mutant hearts which has been previously associated with LVNC.

**Conclusion:**

MIR503HG plays a role in ventricular chamber development, and its deletion leads to an LVNC phenotype independent of the miRNA cluster within its locus, highlighting its importance in cardiac development and disease. We further suggest that abnormal Notch1 activity may underpin the LVNC phenotype presented.

**Time of primary review: 28 days**

The long non-coding RNA (lncRNA) MIR503HG, first described as a precursor for microRNAs (miR)-424 and miR-503, is conserved across species including in humans and mice (Gm28730 in mice), originating from a common tetrapod ancestor. Subsequent studies have shown that MIR503HG has functions distinct from its associated miRNAs, playing important roles in cell proliferation, invasion, apoptosis, and differentiation.^1^ Nonetheless, the shared regulatory regions and overlap with miR-424/503 make the study of MIR503HG function highly challenging. While miR-424/503 are strongly expressed in the developing heart, inducing cardiomyocyte differentiation and inhibiting neuroectodermal cell fate,^2^ the specific role of MIR503HG during development remains completely unexplored.

To investigate this, we generated a MIR503HG functional knockout mouse (*MIR503HG^−/−^*) by introducing a synthetic polyadenylation (SPA) signal leading to premature termination of MIR503HG transcription without disrupting the miRNA cluster (*Figure 1A*). RT-qPCR analysis on 8-week-old *MIR503HG^−/−^* hearts confirmed *MIR503HG* loss (*Figure 1B*) while maintaining *miR-424(322)* and *miR-503* expression (*Figure 1B′* and *B″*).

**Figure 1:**
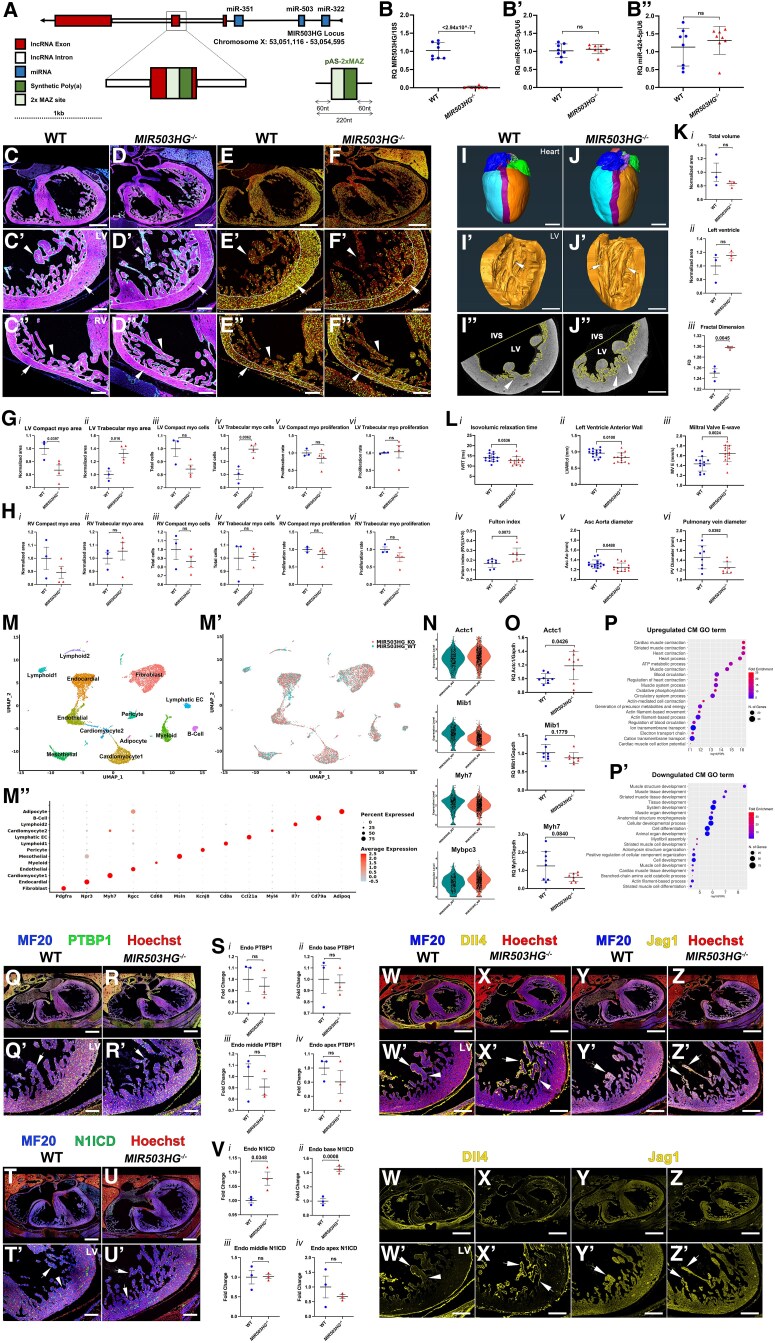
(*A*) CRISPR/Cas9 strategy to generate the *MIR503HG* null allele (*MIR503HG^−/−^*) in mice. To prevent unintended alterations in the gene structure, typical of large deletions, we inserted a SPA signal cassette (pAS-MAZ). This terminates *MIR503HG* transcription while preserving upstream miRNA precursor expression. The SPA cassette was inserted into Exon 1 of the *MIR503HG* gene; this includes a compact and highly efficient SPA followed by two MAZ protein binding sites, which further enhances termination of transcription. This approach ensures specific disruption of MIR503HG lncRNA without affecting miR-322(424)/503 expression. (*B–B″*) Expression of *MIR503HG* (*B*), *miR-503* (*B′*), and *miR-424* (*B″*) in heart tissue from 8-week-old *MIR503HG^−/−^* mice compared to WT littermates was assessed by qPCR. Relative quantification value for gene expression was determined following normalization to the levels of 18S (*n* = 8 mice/group; two-tailed Student’s *t*-test). (*C–F″*) Morphological (*C–D″*) and proliferation (*E–F″*) analysis. Sections underwent immunofluorescence staining for myocardial marker MF20 (1:500, Thermo Fisher, magenta in *C–D″*), endocardial marker CD31 (1:200, DIANOVA, cyan in *C–D″*), proliferation marker Ki67 (1:200, Abcam, green in *E–F″*), and nuclear marker Hoechst (1:1000, Sigma, blue in *C–D″*, red in *E–F″*). Sections show a general view of E17.5 hearts (*C–F*) and a higher magnification view of the left (LV, *C*′–*F*′) and right (RV, *C″–F″*) ventricles from WT (*C–C″* and *E–E″*) and *MIR503HG^−/−^* mutants (*D–D″* and *F–F″*). Arrows point to the compact layer outlined with a white contour; arrowheads point to the trabecular myocardium. (*G–H*) Quantitative analysis of LV (*G*) and RV (*H*) compact myocardium area (i), trabecular myocardium area (ii), compact myocardium cells (iii), trabecular myocardium cells (iv), compact myocardium proliferation (v), and trabecular myocardium proliferation (vi) in *MIR503HG^−/−^* mutants compared with WT (*n* = 4 embryos/group). (*I–J′*) 3D reconstructions from WT (*I*) and *MIR503HG^−/−^* (*J*) adult hearts generated in Amira software (Thermo Fisher) from microCT datasets where right (blue) and left (green) auricles, RV (cyan) and LV (orange), interventricular septum (purple), aorta, pulmonary artery, and tricuspid, bicuspid, and semilunar valves were segmented for morphological analysis and volumetric quantifications. (*I′* and *J′*) 3D reconstructions from WT (*I′*) and *MIR503HG^−/−^* (*J′*) LV showing the trabecular myocardium and papillary muscles from the luminal side of the LV (arrows). (*I″* and *J″*) MicroCT optical transverse sections in the middle region of the LV showing the luminal perimeter used to perform fractal analysis for trabecular complexity. Arrows point to trabecular myocardium. (*K*) Volumetric quantifications of the entire heart (i) and LV (ii) and fractal analysis of LV (iii) (*n* = 3 mice/group). (*L*) Cardiovascular morphology and functional analysis by transthoracic echocardiography on 13-week-old *MIR503HG^−/−^* mutants compared with WT showing quantifications for LV isovolumetric relaxation time (IVRT, i), LV anterior wall thickness (LVAW, ii), mitral valve regurgitation (MV E, iii), Futon index (iv), and ascending aorta (v) and pulmonary vein (vi) diameter. In addition to the structural adaptations described, we identified a decrease in diameter for the ascending aorta and pulmonary vein (v, vi), which may be the result of further changes in cardiovascular development or physiology in mutants (*n* > 8 mice/group; two-tailed Student’s *t*-test). (*M*) Uniform manifold approximation and projection of snRNA-seq) data identifying 14 different clusters at 0.17 clustering resolution, with respective biological cluster identities as defined by canonical marker genes. (*M′*) Analogous uniform manifold approximation and projection displaying both *MIR503HG^−/−^* (red) and WT (green) cell identities suggests no major changes in the cell clusters identified in *MIR503HG^−/−^* hearts compared with WT. (*M″*) Dot plot representation of marker genes for each cluster represented by average expression (colour) and percentage of positive cells (size). (*N* and *N′*) Violin plots showing log normalized expression of *Actc1*, *Myh7*, *Mib1*, and *Mybpc3* in Cardiomyocyte1 cluster. All nuclei used for snRNA-seq analysis were isolated from snap frozen 13-week-old WT and *MIR503HG^−/−^* mutant hearts (*n* = 1 pooling three mice/group). (*O* and *O′*) Validation of snRNA-seq results for *Actc1*, *Myh7*, and *Mib1* expression using qPCR on RNA isolated from snap frozen 13-week-old *MIR503HG^−/−^* and WT whole hearts. Relative quantification values for gene expression were quantified by qPCR assay relative to *Gapdh* (*n* > 8 mice/group; two-tailed Student’s *t*-test). (*P* and *P′*) GO biological process enrichment analysis of up-regulated and down-regulated DEGs in the Cardiomyocyte1 cluster. (*Q–R′* and *T–U′*) Molecular analysis by immunofluorescence of myocardial marker MF20 (1:500, Thermo Fisher, blue), PTBP1 (*Q–R′*; 1:200, Abcam, green), or N1ICD (*T–U′*; 1:100, Cell Signaling, green) and nuclear marker Hoechst (1:1000, Sigma, red) in a mid-ventricular view of a E17.5 heart (*Q*, *R*, *T*, and *U*) and a higher magnification view of LV (*Q′*, *R′*, *T′*, and *U′*) from WT (*Q*, *Q′*, *T*, and *T′*) and *MIR503HG^−/−^* mutants (*R*, *R′*, *U*, and *U′*). (*Q′* and *R′*) Arrows point to PTBP1-positive endocardial cells. (*T′* and *U′*) Arrows point to N1ICD-positive endocardial cells; arrowhead points to N1ICD negative cells. (*S* and *V*) Quantitative analysis of PTBP1 (*S*) and N1ICD (*V*) expression in the entire endocardium (i) and after segmentation of the endocardium into endocardial base (ii), middle (iii), and apex (iv) (*n* = 3 embryos/group). Data in graphs in *B′*, *B″*, *G*, *H*, *K*, *L*, *O*, *S*, and *V* represented as mean ±SEM. Datasets were analysed for normal distribution using Shapiro–Wilk test. Normally distributed datasets were analysed using *t*-test whereas non-normally distributed datasets were analysed using Mann–Whitney test. Statistical significance was considered when *P*<0.05. Significant *P*-values indicated in the graphs; ns, not significant. (*W–Z′*) Molecular analysis by immunofluorescence of myocardial marker MF20 (1:500, Thermo Fisher, blue), Dll4 (*W–X′*; 1:200, R&D Systems, yellow), or Jagged1 (*Y–Z′*; 1:100, Cell Signalling, yellow) and nuclear marker Hoechst (1:1000, Sigma, red) in a mid-ventricular view of a E17.5 heart (*W–Z*) and a higher magnification view of LV (*W′–Z′*) from WT (*W*, *W′*, *Y*, and *Y′*) and *MIR503HG^−/−^* mutants (*X*, *X′*, *Z*, and *Z′*). Arrows point to Dll4 or Jagged1 positive cells (*n* = 3 embryos/group). Scale bars: 200 µm in *Q-R, T-U and W-Z*; 100 µm in *C–F*; 50 µm in *C′–F″*, *Q′–R′*, *T′–U′*, and *W′–Z′*; 2 mm in *I–J*; 1 mm in *I′–J′*, and *I″–J″*.

Morphological analysis on embryonic heart sections of *MIR503HG^−/−^* mutants compared to wild type (WT) revealed no defects in endocardium or myocardium of the atria, atrioventricular canal (AVC), or left ventricle (LV) at E10.5 (not shown). Similar findings were observed for the right atrium and AVC valves at E17.5 (not shown). However, a notable reduction in compact myocardium thickness and an increase in trabecular myocardium were observed in *MIR503HG^−/−^* mutant LV compared to WT at E17.5, while the right ventricle (RV) remained unaffected (*Figure 1C, D″*, *G*, and *H*). Trabecular myocardium forms from the compact layer cardiomyocytes until E14.5, when the process of ventricular compaction is initiated, whereby trabeculae become incorporated into the compact layer and contribute to thickening of the ventricular wall.^3^ Abnormal ventricular compaction leads to left ventricular non-compaction (LVNC), characterized by an increase in trabecular myocardium and a thinning of compact myocardium. These defects can be due to changes in cell number or cell size, whereas changes in cell number can be due to proliferation defects or differential allocation of cells to trabecular or compact layers. Cell quantifications found a statistically significant increase in trabecular cells in LV, with no changes in RV (*Figure 1G* and *H*). Analysis of proliferative competence using Ki67 expression showed no obvious differences in any of the tissues forming *MIR503HG^−/−^* ventricles compared to WT (*Figure 1G* and *H*). These results suggest that *MIR503HG^−/−^* LV defects are associated with abnormal ventricular compaction.

Analysis of chamber and septal tissue volumes in adult hearts by microCT (*Figure 1I–K*) did not reveal any significant defects (*Figure 1I* and *J*). However, the trabecular myocardium and papillary muscles of the LV appeared more complex in *MIR503HG^−/−^* mutants compared to WT (*Figure 1I′*, *J′*, and *K*). Fractal analysis revealed a significant increase in trabecular complexity in *MIR503HG^−/−^* LV compared to WT, consistent with a LVNC phenotype (*Figure 1I″*, *J″*, and *K*). Cardiac functional analysis via transthoracic echocardiography identified impaired LV relaxation, a thinner LV anterior wall, and mitral valve (MV) regurgitation in *MIR503HG^/−^* mutants (*Figure 1L*). Notably, LV contractile dysfunction and MV regurgitation are reported in LVNC patients. Despite these changes, no premature mortality in *MIR503HG^−/−^* mutants was observed, suggesting possible compensatory mechanisms, although more severe phenotypes may develop with age.

To identify molecular defects associated with the LVNC phenotype in *MIR503HG^−/−^* mutants, we performed single-nuclei RNA sequencing (snRNA-seq) on 13-week-old adult WT and *MIR503HG^−/−^* hearts after pooling three individual samples per condition (*Figure 1M*, *M′*, and *M″*). Analysis of differentially expressed genes (DEGs) suggested changes in *Actc1*, *Mib1*, *Mybpc3*, and *Myh7* expression in cardiomyocytes in *MIR503HG^−/−^* mutants (*Figure 1N*), as well as *Myl2*, *Mb*, *Myl3*, *Tnnt2*, *Tnni3*, and *Actc1* expression across multiple cell clusters (not shown). Notably, mutations in *ACTC1*, *MIB1*, *MYBPC3*, *MYH7*, and *TNNT2* have been associated with LVNC,^4^ supporting the classification of *MIR503HG^−/−^* cardiac defects presented here. Changes in *Actc1*, *Mib1*, and *Myh7* expression were further validated by qPCR (*Figure 1O*). Moreover, Gene Ontology (GO) analysis of DEGs in cardiomyocytes, both up- and down-regulated, highlighted terms associated with cardiac muscle contraction and cardiac muscle structure development (*Figure 1P* and *P′*).

We have previously shown that MIR503HG directly interacts with the alternative splicing factor PTBP1 to regulate endothelial function.^1^ Whereas it is interesting that *Ptbp1* deletion also leads to LVNC, expression of PTBP1 in *MIR503HG^−/−^* mutants was not affected (*Figure 1Q–S*). Nonetheless, further analysis would be needed to assess whether PTBP1 activity is affected in *MIR503HG^−/−^* mutants. Abnormal NOTCH pathway activity is also associated with LVNC, including reduced expression of *Mib1*, encoding an ubiquitin ligase critical for Notch ligand endocytosis and Notch signalling (*Figure 1N* and *O*). Notch1 intracellular domain (N1ICD) immunofluorescence, a readout of Notch1 activity, identified a significant increase in N1ICD expression in endocardial cells at the base of trabeculae in *MIR503HG^−/−^* mutants at E17.5 (*Figure 1T–V*). Expression analysis of Notch pathway ligands identified an increase in Jagged1 myocardial expression and an expansion of Dll4 expression towards the trabecular base, which are likely responsible for the increased N1ICD expression (*Figure 1W–Z*). LVNC has been reported in a mouse model with excessive Notch1 activity,^5^ suggesting that abnormal Notch ligand expression and Notch1 activity may underpin the LVNC phenotype in *MIR503HG^−/−^* mutants. Further research is needed to identify how the Notch pathway is regulated by MIR503HG.

In summary, our findings suggest that MIR503HG may play an important role in ventricular chamber development and in the pathogenesis of LVNC independently of the miRNA clusters associated with its genetic locus.

All studies were approved by the University of Edinburgh Committee Board. All animal experiments were performed in accordance with the Animals (Scientific Procedures) Act (UK) 1986 and under the auspices of UK Home Office Project and Personal Licenses (number PP3528002) held within The University of Edinburgh. Each mouse was anaesthetized using 3% (v/v) isoflurane (Abbot Laboratories, Berkshire, UK) supplemented with O_2_ at a flow rate of 0.5 L/min. At the completion of recording, all mice were euthanized by a Schedule 1 method, under terminal isoflurane anaesthesia. Data supporting the study are available from the corresponding authors on request.
